# Neuralgic Headache

**Published:** 1854-08

**Authors:** J. Murphy


Neuralgic Headache. By J. Murphy, M. P.—Neuralgic headache is synonymous with those headaches described by some old authors as hemicrania, by others as clavus hystericus, and by Dr. Graves as hysterical congestion. It is *peculiar* to females, and to females during a certain period of their existence only, from puberty until the final cessation of the menstrual secretion. Dr. Graves gives a graphic description of the symptoms and of the injurious effects of the usual routine of treatment. He calls it hysterical congestion; but he seems not to have understood its true pathology. There is no doubt of its being hysterical; but there is no congestion, for the seat of the pain is in one of the nerves of the scalp, which can be easily proved by a slight examination, and it is therefore an external headache. The error may have arisen from his having met with cases where this headache was in combination with the anæmic headache. The proper name which should be bestowed on this headache, in order to facilitate the diagnosis, is spinal irritation of the sub-occipital nerve. Spinal irritation is beginning to be well understood in this country; we are indebted to a French physician, M. Valleix, for the discovery. Since then many other disorders, such as irritable mammæ, pleurodyne, and neuralgic headache, are discovered to originate in functional derangement of the spinal cord; and I believe whoever will carefully compare these disorders with cases related by Dr. Tilt, must come to the conclusion that they are nothing more than symptoms of subacute ovaritis. They are hysterial disorders, and *hysteria is subacute ovaritis*, which displays its phenomena on the sensitive and motive nerves of the spinal column.

On comparing the neuralgic headache with the phenomena of spinal irritation in other parts, we find how exactly they coincide. Like spinal irritation, it is a form of hysteria, and therefore peculiar to females. It is not only peculiar to females, but attacks them only during the menstruating period of their existence; that is, from about the thirteenth to the fiftieth year. It is exacerbated just previous to menstruation, makes its first attack on the left side, and rarely passes over to the right side.

*Cause.*—As this form of headache is peculiar to the female sex it must therefore have its origin in some organ peculiar to them; and as it is felt during a certain period of existence only, the organ must have the performance of its functions limited to that period. As there is no organ by which these two facts are explicable, unless the ovarium, it is not unphilosophical to conclude that the disorder proceeds from the ovarium. There is certainly also the uterus, but the functions of this viscus cease on the removal of the ovaria. We daily meet with the uterus inflamed, ulcerated from cancer or cauliflower excrescences, distended by hydatids or pregnancy, producing moles and polypi, but none of the phenomena of spinal irritation are present. In the married female, who bears children regularly, it is scarcely ever known. Before the commencement of menstruation, or after its termination, it is equally rare.* What is the state of the ovarium, I do not pretend to affirm. If inflammation, yet it has often yielded to tonics; it may depend no moral causes, but such an explanation has never satisfied me. An accumulation of fœces in the rectum has appeared to me as occasionally the source of irritation; in a few cases, I think it was traceable to ascarides in the rectum. We witness the action of cold in paralyzing the trunk of a motor nerve, the portio dura, as it escapes from its cranial foramen; but cold cannot be a cause of this headache, otherwise why should not the male sex equally suffer.

Occasionally, spinal irritation, in other parts, has been observed earlier in life, but I have not met with the headache; and, as the headache has occurred some years before the appearance of the menses, so I believe it possible it may arise a few years after their total cessation. The headache resembles spinal irritation, also, in a curious and hitherto unexplained phenomena; *commencing* on the left half of the body, we occasionally met with it also on the

---

* While writing the above, I referred to Dr. Tilt's work on Diseases of Females, first edition, and on page 58, he gives the valuable fact that he found the right ovary affected in only five out of seventeen cases. Now, it might be worth inquiry to ascertain whether the left had not been previously affected, but that the irritation was transferred to the right, as we see in ophthalmia occasionally.

other side; but I have never discovered that it began there, nor is it ever restricted solely to that side. When both sides are attacked they are equally so, the left being by far the more painful. As another proof of its being spinal irritation, if further proof be necessary, we find it under two distinct forms, and these forms are easily distinguishable by the nature and extent of the pain. In the one, it is confined to the exact tract of the sub-occipital nerve, it is lancinating or shooting, intermitting, and chiefly felt at its termination in the integuments of the temporal region; when severe in this spot it is the *clavus hystericus.* When the whole course of the nerve and its branches are implicated, the entire left side of the scalp is very tender, sometimes exquisitely so; this is the hemicrania. It is singular how much this disease is confined to the left side of the head; we find such to be the fact in ninety-nine cases out of a hundred. It seldom reaches the aggravated form of *clavus hystericus* without being accompanied with other well-known hysterical symptoms, which of course, facilitate the diagnosis.

*Diagnosis.*—This headache attacks females exclusively. I have never heard or read of men suffering from this kind of headache. It is only during the menstruating period of life that even females are liable. The pain is referred to the left side of the head; it is worse on the approach of the menstrual flow; it is found in the track of the sub occipital nerve. The course of this nerve is well known; it accompanies the sub-occipital artery, emerging from the spinal canal; it passes along the back of the head, midway between the mastoid process and the mesial line, sending branches to the integuments which cover the parietal protuberance, and terminating in the temporal region. Its course, from its exit to its termination, can oftentimes be accurately ascertained, from the pain induced by pressing upon it. Although the head suffers, pressure may not always produce the pain, for it is intermitting. In general, however, pain may be thus detected in one of three places; on the left side of the neck, where the head and vertebræ join, at the parietal protuberance, or in the temporal region; when concentrated in the last spot, it is the well-known clavus. It is sometimes painful in all three, and sometimes in its whole track. It is, however, rare that the tenderness is absent in the occipital region. The part suffers more when pinched than when pressed. When the branches as well as trunk suffer, we then have hemicrania, a most painful form, less intermitting than the other, and preventing the unfortunate female from lying on the affected side. It is more commonly met with in the unmarried female, from the twenty second to the thirty-fifth year; but the married females who are childless do not escape.

This headache is chronic, intermitting, may continue for days, weeks, or months, then subside, and return after a lapse of months or even years. A first attack is seldom felt before the twentieth year, nor after the thirty-fifth. The pain is generally of a shooting kind, darting from the neck towards the temple, and never towards the neck, by which it is easily distinguished from odontalgic pain. Neuralgia of the left mamma (irritable breast), or of the seventh or eighth intercostal (pleurodyne), frequently coexists. It is sometimes found in combination with the anæmic, but more rarely with the congestive headache. From caries of the body of a vertebra it is easily distinguished by the pain being superficial, being confined to the left side of the spine, by its not becoming worse when the head is flexed on the chest, nor by jumping, nor by pressing the head against the spinal column.

This neuralgic pain sometimes accompanies the rotated spine. It is singular how often toothache is mistaken for headache, especially for this form. In both, the pain is described as shooting in the course of the nerves but in toothache the pain shoots towards the neck and ear, leaves no tenderness of scalp, never goes so high as the parietal protuberance, and is more correctly discovered by learning that a paroxysm is brought on by food, sometimes when warm, at other times when cold.

*Treatment.*—If the disease be not complicated, we can promise relief. The bowels should be kept open by regulated diet, and by aperients, such as caster oil, olive oil, lenitive electuary, powdered rhubarb, soluble tartar, or the compound rhubarb pill. If the bowels are obstinate, an enema of a pint of cold water daily answers the double purpose of removing the contents which may irritate the ovary, and as a local application to the organ chiefly in fault. The cold hip-bath is a valuable remedy when the constitution is vigorous, but all these things are inferior to sea-bathing. Stimulants should be abstained from, employment should be found for mind or body, but physical efforts are preferable. The sedentary position required by the needle, especially in solitude, is very injurious. A sinapism over the exit of the nerve gives great temporary relief; a vesicating plaster of cantharides is better, but it oftentimes leaves a mark, and therefore, on account of sex, age, and position in life, may be objectionable. A croton-oil liniment, made with one drachm of oil and one ounce of camphorated tincture of opium, and rubbed until pustules appear, is preferable, as it leaves no permanent blemish. The belladonna plaster, mixed with powdered opium, or a liniment of extract of belladonna, rubbed with mucilage, are useful and unobjectionable remedies.—Speedy relief is occasionally afforded by veratrine or aconitine ointment, made with from four to six grains to half an ounce of spermaceti ointment. The finger used in rubbing should have a piece of bladder interposed.

One ounce of tincture of aconite, with seven ounces of rosewater, is a safer remedy to trust to inexperienced hands than the veratria. The internal medicines are not so easily chosen. Tonics are frequently required, and they may be combined with anti-hysteric remedies. The bisulphate of quinia may be exhibited in a strong infusion of valerian; compound iron pill, with assafetida in large doses, is very beneficial. If there be irritability of the stomach co-existing with profuse menstruation and leucorrhœa, pills of valerianate of zinc, half a grain three times a day, with one drop of creasote, answer many intentions. If there be much debility, the sulphate of iron may be combined with infusion of valerian and ammonia, or the ammoniated tincture of valerian may be prescribed. The pain is sometimes so acute that some relief is quickly demanded, and half a grain of morphia will lessen the pain for a while until other remedies have time to act. For the leucorrhœa, one drachm of acetate of zinc to one pound of distilled water is useful as a lotion. But we are sometimes perplexed, for the tonic treatment is not the best for a full plethoric female; leeching or even general bleeding is required, but the cases are rare which require general bleeding.

If the patient be not very weak, and there is much leucorrhœa and memorrhagia, the treatment laid down by Dr. Tilt for subacute ovaritis should be adopted. He leeches in the menstrual interval, and then blisters the iliac regions; but as his work is universally read, the treatment is well known. Sea-bathing, when practicable, should never be omitted.—*London Lancet*, May 20, 1854.

---

Acetate of Iron, for Aneurism.—*Radical Cure of an Aneurism of the External Maxillary Artery, by the Injection of the Acetate of Iron:* By Dr. Filippo Lussana, of Milan.—Antecedent to the experiment of Pravaz, a celebrated Italian surgeon, Monteggia, had suggested in the treatment of aneurisms "the injection of astringents into the sac, after entering it with a trocar, in order to obtain a prompt and durable coagulation of the blood, the artery being compressed above the tumor." (See *Istituzioni Chirurgiche*, vol. ii, 2d ed., Milan.) The idea of this operator was never carried out, however, and it remained for the skilful and persevering Pravaz to introduce this great improvement into the practice of surgery. Soon after the researches of Pravaz were made known, and the use of the perchloride of iron in aneurisms had been followed by some disasters, Dr. Ruspini commenced a series of experiments with a view of substituting some less deleterious agent for the perchloride of iron. He finally employed the acetate of the sesquioxide of iron, a salt which contains no principle injurious to the economy, and which possesses great hæmostatic power. This agent had only been employed in cases of slight hæmorrhage and in experiments on animals, when Dr. Lussana determined to employ it in an operation on an aneurism. His experiment resulted most successfully.

M. Gelmi, a girl of twenty-two, presented an aneurismal tumor of the size of a large walnut, between the corner of the mouth and angle of the lower jaw. This tumor was punctured with a small lancet; arterial blood immediately issued in jets. A fine syringe was introduced, and ten drops of the ferruginous solution was injected. The hæmorrhage and pulsations were immediately arrested. The operation caused but little pain and no inflammation succeeded it. In ten days the tumor had sensibly contracted. An exploratory puncture was made in it without causing any hæmorrhage.—*Va. Med. and Surg. Jour.—From Gaz. Méd. de Paris, of Feb.* 25*th*, 1854.

